# Invasion and Colonization of Pathogenic *Fusarium oxysporum* R1 in *Crocus sativus* L. during Corm Rot Disease Progression

**DOI:** 10.3390/jof8121246

**Published:** 2022-11-25

**Authors:** Nancy Bhagat, Shanu Magotra, Rikita Gupta, Shikha Sharma, Sandhya Verma, Praveen Kumar Verma, Tahir Ali, Ankita Shree, Jyoti Vakhlu

**Affiliations:** 1 Metagenomic Laboratory, School of Biotechnology, University of Jammu, Jammu 180006, India; 2 University Institute of Biotechnology, Chandigarh University, Ajitgarh 140413, India; 3 Plant Immunity Laboratory, National Institute of Plant Genome Research (NIPGR), New Delhi 110067, India; 4 School of Life Sciences, Jawaharlal Nehru University, New Delhi 110067, India

**Keywords:** *Crocus sativus*, *Fusarium oxysporum*, corm-rot of saffron, molecular identification, housekeeping genes

## Abstract

The corm rot of saffron caused by *Fusarium oxysporum* (Fox) has been reported to be the most destructive fungal disease of the herb globally. The pathogen, *Fusarium oxysporum* R1 (Fox R1) isolated by our group from Kashmir, India, was found to be different from *Fusarium oxysporum* f.sp. *gladioli* commonly reported corm rot agent of saffron. In the present study, Fox R1 was further characterized using housekeeping genes and pathogenicity tests, as *Fusarium oxysporum* R1 f.sp. *iridacearum* race 4. Though Fox R1 invaded the saffron plant through both corm and roots, the corm was found to be the preferred site of infection. In addition, the route of pathogen movement wastracked by monitoring visual symptoms, semi-quantitative PCR, quantitative-PCR (q-PCR), real-time imaging of *egfp*-tagged *Fusarium oxysporum* R1, and Fox R1 load quantification. This study is the first study of its kind on the bidirectional pathogenesis from corm to roots and vice-versa, as the literature only reports unidirectional upward movement from roots to other parts of the plant. In addition, the colonization pattern of Fox R1 in saffron corms and roots was studied. The present study involved a systematic elucidation of the mode and mechanism of pathogenesis in the saffron *Fusarium oxysporum* strain R1 pathosystem.

## 1. Introduction

*Crocus sativus* L. (Saffron) is a low-volume, high-value perennial crop, cultivated for its red stigmas [1,2,3,4]. It is the world’s costliest spice with one kg costing around 11,000 USD [5,6]. Iran is the largest producer of saffron that accounts for approximately 90% of global production followed by India and Greece [7,8]. Jammu and Kashmir (32°17′ and 36°58′ north latitude and 37°26′ and 80°30′ east longitude) in the North-Western Himalayas, is the only region with the distinction of cultivation of this spice in India at a commercial scale [1,9,10]. Saffron cultivation has been severely affected by the corm rot disease caused by *Fusarium oxysporum* the world over [8]. 

The flowering stage in the life cycle of saffron has been considered to be the most vulnerable stage for Fox infection. The pathogenic fungus invades the plants through mycelium or the germ tube of chlamydospores [7]. Once infected, this infection is transferred from the mother corms to the daughter corms resulting in a decrease in the production of flowers, and a number of daughter corms with a reduced weight that results in severe yield losses [11]. For the process of invasion, Fox elicits many hydrolytic enzymes and mycotoxins. In 2020, Khaledi and co-workers reported the association of cell wall degrading enzymes released by *Fusarium oxysporum* with the root and corm infection of saffron in south Khorasan province [12]. In India, according to an estimate (1999–2000) the disease incidence ranged from 42.6–59.3% in the Kishtwar district and 4.6–42% in saffron cultivating regions of Kashmir resulting in major yield loss [7]. The production of saffron declined drastically due to the use of disease corms for propagation, i.e., 15.95 Mts in 1997 to 9.6 Mts in 2015 to 3.83 Mts in 2022 [13].

Corm rot results in spongy decay, deformations, and discoloration of corms that ultimately leads to wilting of the leaves and death of the plant [14,15,16,17,18,19]. *Fusarium oxysporum* (corm rot agent) from saffron in Italy and Spain has been classified as *Fusarium oxysporum* f.sp. *gladioli* [20] but the *Fusarium oxysporum* (Fox) pathogen of saffron from Kashmir and Kishtwar has not been classified up to *forma specialis* level [16,21]. 

*Fusarium oxysporum* R1 was isolated from the rotten corms collected from the Pampore region of Kashmir and its pathogenicity was confirmed by Koch postulates [16]. In our previous study, based on ITS marker phylogeny, Fox R1 was found to be different from Fox f.sp. *gladioli*; the commonly reported saffron pathogen [15,16]. Therefore, in the present study, Fox R1 was classified based on a comparison of selected housekeeping gene sequences and disease assays. 

In other plants, Fox is reported to penetrate roots asymptomatically and then colonize vascular tissue, setting off chlorosis, wilting and necrosis of aerial plant parts [22,23,24]. *Fusarium oxysporum* multiplies in infected tissues by forming conidia (microconidia and macroconidia), mycelium as well as chlamydospores [21,25,26]. However, in the case of saffron, the exact mechanism by which Fox infects and colonizes remains poorly understood. Recent advancements in imaging techniques have enabled a better understanding of the plant–pathogen interplay [27,28]. Detailed understanding of the in-vivo interactions between the pathogenic fungus and the saffron, i.e., by imaging and molecular approaches could lead to the discovery of efficient ways to control this disease. 

Since the information on the site of infection and pattern of colonization during corm rot disease in saffron and other corm-bearing plants is lacking so, the present study was undertaken to unravel the primary route of infection during Fox R1 invasion on saffron, in addition to the classification of the pathogenic Fox R1 up to *forma specialis*. To our knowledge, this is the first study on the invasion, infection, and colonization of *Fusarium oxysporum* in saffron. 

## 2. Materials and Methods

### 2.1. Corm Sample, Fungal and Bacterial Strains and Culture Conditions

**Figure 1:**
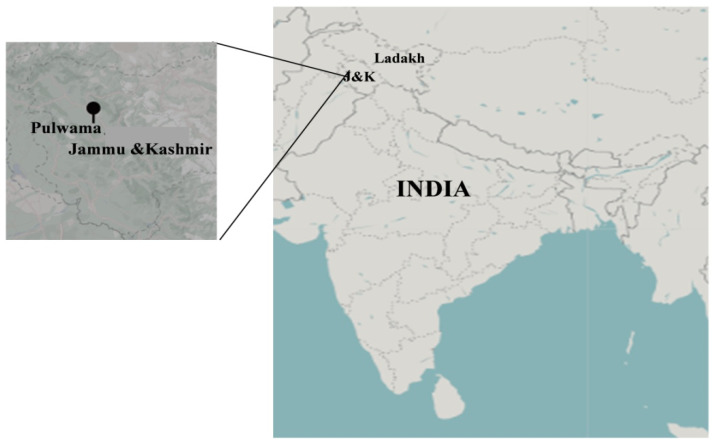
Map representing sample collection site.

Saffron corms were collected from the Pulwama district of the Kashmir region of Jammu and Kashmir during its dormant phase in August 2019 (Figure 1). The pathogenic *Fusarium oxysporum* R1 (Fox R1) used in the present study, has been reported earlier and was isolated from the rotten corms from Pulwama, Kashmir region (34°01′12.00′′ N 74°55′48.00′′ E) [16]. The fungal culture was cultivated on potato dextrose agar plates and slants (Difco, BD Becton, and Dickinson) at 25 °C for 5 days in the incubator (ORBITEK). Fox R1 spore suspension of 10^12^ spores/mL was prepared as per the protocol developed earlier by our group [16].

*Agrobacterium tumefaciens* strain GV3101 with *egfp* gene containing plasmid [29] was used for the transformation of Fox R1. The bacterial culture was grown in Luria Bertani broth supplemented with 100 µg/mL kanamycin and 30 µg/mL rifampicin, at 28 °C and 150 rpm for 24 h [30].

### 2.2. Molecular Characterization of Fox R1

Genomic DNA was isolated using the DNA isolation method as described by [31]. The primers reported in previous studies for housekeeping genes such as translation elongation factor 1α (*EF-1α*), β-tubulin (*Bt*), histone 3 (*H3*), actin (*Act*), and calmodulin (*Cal*) was used to characterize Fox R1 [32,33,34,35]. An amount of 5–20 ng of genomic DNA per 10 µL of PCR reaction volume was used and the PCR amplification was performed as described earlier [16]. Sequencing of PCR products was completed from SciGenom Labs Private Ltd., Cochin; Kerala, India. Amplicon sequences thus obtained were subjected to the BLASTn sequence similarity search tool for phylogeny study [36]. These sequences were submitted to the GenBank database and phylogenetic trees were constructed using the Maximum Composite Likelihood model and bootstrap test (1000 replicates) in Mega X software (https://www.megasoftware.net/ (accessed on 5 April 2022).

### 2.3. Disease Assays for Fox R1 Characterization

To determine *forma specialis* of Fox R1, disease assays were performed on four plant species belonging to the Iridaceae family that included *Sparaxis* sp., *Tritonia* sp., *Gladiolus* sp., and *Ixia* sp. Saffron corms were taken as a control for disease assay. Corms and seeds sown in sterile soil in pots were infected with the pathogen using a sterile syringe needle. Corms without any treatment were taken as a negative control. All the assays were performed in triplicates and corms and seeds were observed till 15 and 20 days post-inoculation (dpi). Fungal isolation was performed from the infected corms and roots to confirm the identity of the pathogen based on morphology, microscopy, and ITS phylogeny. 

### 2.4. Experimental Design to Study the Route of Infection of Fox R1 in Saffron Plant

**Figure 2:**
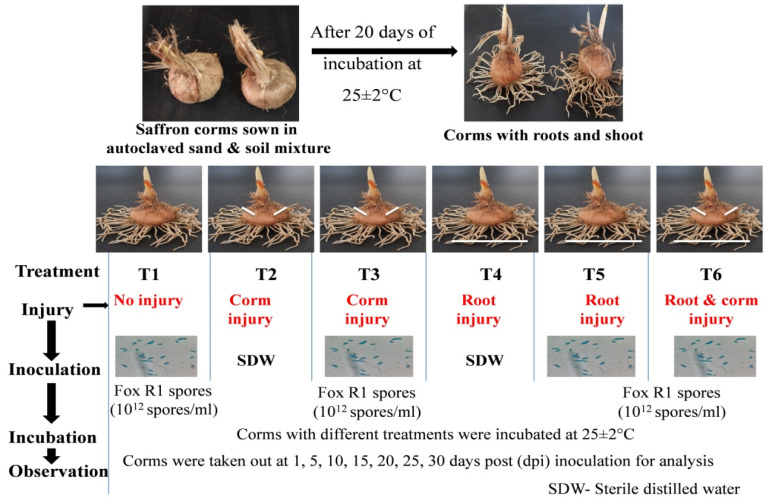
Experimental layout to study the route of infection of *Fusarium oxysporum* R1 (Fox R1) in the saffron plant.

To study the route of Fox R1 invasion in the saffron plant, an experiment was performed in a complete randomized block design (CRDB). Corms were grown in pots (pots of 76 mm mouth diameter) containing steam-sterilized sand: soil mixture (1:1 *w*/*w*). These pots were incubated for 20 days at 24 °C (12 h light/dark intensity of light) in the plant growth chamber for root initiation and growth. After 20 days, growing corms were taken out and treated variously (Figure 2). Corms were injured with a sterile needle (1 cm deep) and two injuries were given to each corm. Roots were injured by cutting the root tips (0.2 cm) with sterile scissors. Disease progression in both corms and roots was observed at 1, 3, 5, 10, 15, 20, 25, and 30 days post-inoculation. Each treatment was laid in 5 replicates. 

The details of the different treatments are as follows: (T1) roots and corms without injury but Fox R1 spore suspension was added to pot soil. (T2) Corm injury (CI) but Fox R1 spore suspension was not added to pot soil. (T3) Corm injury (CI) and Fox R1 spore suspension were added to pot soil. (T4) Root injury (RI) but Fox R1 spore suspension was not added to pot soil. (T5) Root injury (RI) and Fox R1 spore suspension were added to pot soil. (T6) Roots and corm injury (RCI) and Fox R1 spore suspension was added to pot soil. The graphical representation of different treatments given to the saffron plant has been shown in Figure 2. 

### 2.5. Confirmation of Infection and Movement of Fox R1 by Semi-Quantitative PCR and q-PCR

#### 2.5.1. Designing of Primer and Sensitivity of Primers

To check the presence and quantify Fox R1 at the infection site (corms and roots) and to investigate its movement from corm to roots and from roots to corm, Fox R1-specific primers were designed. The Fox R1 ITS gene sequence was used for primer designing. Ten different primer pairs were designed using the primer blast tool (https://www.ncbi.nlm.nih.gov/tools/primer-blast/ (accessed on 10 August 2021). All the pairs were checked for cross-amplification with corm DNA in PCR assay. The primer pair (ITS gene with sequence forward 5′-GAACGCACATTGCGCCCGCCAGTA-3′ and reverse 5′-CGCGAATTAACGCGAGTCCCAACA-3′) was selected based on its specificity to Fox R1 and with no cross-amplification with corm to amplify Fox R1 ITS gene in infected tissue. 

The sensitivity of the primer pair was determined by 10-fold serial dilution prepared in TE buffer of the Fox R1 genomic DNA (100 ng^−1^ fg). The different dilutions were further used for the formation of a standard curve using q-PCR. The q-PCR was performed in 8-well strips using SYBR Green-based assay (Thermo-Scientific, Waltham, MA, USA) on a 7500 Real-Time PCR System (Applied Biosystems^®^, Waltham, MA, USA). The total reaction mixture (10 μL) consists of SYBR Green Master Mix (5 μL), DNA template (1 μL), and gene-specific primers (0.5 μM each). PCR program for amplification was as follows; holding stage at 95 °C for 10 min, followed by 40 cycles at 95 °C for 15 s, 60 °C for 1 min, 72 °C for 30 s, and final stage at 95 °C for 30 s, 60 °C for 15 s, 72 °C for 30 s. Non-template reactions were used as control. The reaction was performed in three replicates. The standard regression equation was used for the conversion of Ct values. The efficiency of the q-PCR reaction was determined using the formula E = (10(−1/slope) − 1) × 100%. 

#### 2.5.2. In Planta Detection and Quantification of Fox R1 in Infected Roots and Corms by Semi-Quantitative PCR and q-PCR

The genomic DNA was isolated from infected roots and corms using the CTAB method [37]. The part of roots and corms used for the isolation of DNA from different treatments has been tabulated in Table 1. The quality of extracted DNA was checked using agarose gel electrophoresis and concentration and purity were checked using Nanodrop (Thermo Fischer Scientific, Waltham, MA, USA). The Fox R1-specific primers were used for the amplification of Fox R1 in infected tissue. The PCR program used for the detection of Fox R1 by semi-quantitative PCR was as follows: DNA was denatured at 94 °C for 5 min as the initial denaturation step, followed by 30 cycles of denaturation at 94 °C for 1 min, annealing at 60 °C for 1 min and extension temperature was 72 °C for 1 min. The final extension was at 72 °C for 7 min. The amplified products were loaded on 1.5% agarose gel and a 100 bp ladder (Himedia, Mumbai, India) was used as the marker. 

For the in planta quantification of Fox R1 by q-PCR and to assess the detection limit, the standard curve was drawn using 10-fold serially diluted DNA. The q-PCR was performed as mentioned above. Non-template reactions were used as control. The reaction was performed in three replicates. The concentration of unknown samples was calculated using the formula 10^((Cq−b)/m)^, where b is the intercept and m is the slope of the regression equation (https://nebiocalculator.neb.com/#!/qPCRlibQnt (accessed on 25 September 2022).

### 2.6. Calculation of Fox R1 Load at the Site of Infection 

The Fox R1 load was determined by the colony forming unit (CFU) method. Komada medium (*Fusarium* specific) was used for the cultivation of Fox R1 from infected sites (described in Table 1) [38]. An amount of 100 mg of the tissue was taken and washed with sterile distilled water inside the laminar airflow to maintain sterile conditions. Infected tissue was crushed using sterile pestle mortar and then 1 mL of distilled water was added. Serial dilution of the suspension was completed, and 10^−2^ dilution was spread on the media plates and incubated at 28 °C for 5 days. After 5 days, colonies were counted, and the load was determined. The Fox R1 colonies obtained were randomly selected and identified by light microscopy and PCR. DNA was isolated using the CTAB method and PCR was performed using Fox R1-specific ITS primers as described in Section 2.7. The load was determined using 5 replicates of each treatment. 

### 2.7. Agrobacterium Tumefaciens Mediated Transformation (ATMT) of Fox R1

Wild-type Fox R1 was cultured on different concentrations of hygromycin B to determine its resistance level. PDA plates containing varying concentrations of hygromycin B (20, 40, 60, 80, and 100 µg/mL) were prepared and an agar plug from 5 days old Fox R1 culture was transferred to the plates. The plates were incubated at 25 °C for 8 days. Fox R1 transformation was performed using the protocol given by [39] with some modifications. Modifications in brief are, *A. tumefaciens* strain GV3101 transformed with pBIF-EGFP vector [30] was cultured on YEP agar plates supplemented with 30 µg/mL rifampicin and 100 µg/mL kanamycin at 28 °C for 2 days. After 2 days, a single colony was picked and further inoculated in YEP broth containing 30 µg/mL rifampicin and 100 µg/mL kanamycin at 28 °C, 150 rpm, and was grown until the OD_600_ reached up to 0.5–0.8. Cells were harvested at 5000 rpm for 5 min at room temperature and washed two times with one volume of minimal medium [40]. After washing, the cell pellet was diluted in 25 mL of induction medium (IM) [40] with 0.2 mM concentration of acetosyringone to obtain an OD_600_ of 0.15 followed by incubation at 28 °C for 6 h at 150 rpm until the OD_600_ reached 0.3–0.4. Fox R1 spores were harvested from 8 days old mycelium grown on PDA and filtered using Miracloth, washed twice with 1 mL of IM, and re-suspended in IM to a final concentration of 1 × 10^6^ or 1 × 10^7^ spores/mL. Finally, 100 µL of IM-suspended *Agrobacterium* culture was mixed with 100 µL of Fox R1 spore suspension. The suspension mix (200 µL) was spread on Petri plates containing IM agar and sterile cellophane discs and co-cultivated for two days at 25 °C. The cellophane discs were then transferred to PDA plates containing hygromycin B (100 µL) and 200 µM cefotaxime and incubated at 25 °C. Subsequently, transformants were transferred to PDA plates containing 100 µg/mL of hygromycin B [39]. 

### 2.8. Molecular Characterization of Transformed Fox R1

Randomly, five enhanced green fluorescent protein transformed Fox R1 isolates were grown in 100 mL PDB containing hygromycin B (100 µg/mL) at 25 °C, 150 rpm for 8 days. Mycelium mass was collected through muslin cloth and was crushed to a fine powder using liquid nitrogen. DNA was isolated using Quick-DNA^TM^ Fungal Miniprep Kit (Zymo Research, Irvine, CA, USA) according to the manufacturer’s instructions. PCR amplification was performed using a specific primer set viz PgpdA F and TtrpC R. The number of T-DNA insertion events throughout the transformed host genome was determined by Southern blot analysis. The PCR-amplified EGFP fragment was eluted from the gel and radio-labeled with [α-^32^P] dCTP using the NEBlot Kit (NEB, Ipswich, MA, USA) according to the manufacturer’s instructions. Radio-labeled amplicons were used as probes. Extracted DNA (8 µg) was digested with *Eco*RI, and the products were separated by electrophoresis on a 0.7% agarose gel in 1XTAE. The gel was treated with 0.25 M HCl before blotting onto a nylon membrane (Amersham Biosciences, Amersham, UK). Denaturation, transfer, pre-hybridization, hybridization, and high stringency washes for Southern blot analysis were carried out using standard protocols [41]. 

### 2.9. Microscopic Monitoring of Transformed Fox R1

To visualize the fungal spore germination events and to determine in planta colonization fluorescent protein expression was studied by an inverted laser-scanning confocal microscope, Leica TCS SP8 AOBS (Leica Microsystems, Exton, PA, Wetzlar, Germany). To study the expression of EGFP in transformed Fox R1, spores were harvested from PDA plates and placed onto a glass slide, and incubated at 25 °C for seven to eight days under high humidity conditions and spores were monitored at regular intervals of times.

### 2.10. Disease Progression in Saffron Infected Corms and Roots 

Saffron corms were sown in pots for 20 days for acclimatization and root initiation at 24 °C (12 h light/dark) before infection. To study the infection pattern of Fox R1 in saffron, six-day-old culture was utilized for the preparation of spore suspension. The saffron corms and roots, already sown were taken out and given injury with a sterile needle. The spore suspension (1 × 10^12^ spores/mL) was mixed with the soil and the pots were incubated again at 24 °C (12 h light/dark). To study the colonization pattern of Fox R1, microscopic examination of corm was performed at 6 hpi, 12 hpi, 1, 2, 3, 4, 5, 8, 12, and 20 days post inoculation (dpi) and of roots at 12 hpi, 1 dpi, 5 dpi, and 10 dpi. Corms were carefully taken out from pots and rinsed gently with distilled water to remove any soil adhered to the corm surface and roots. All the experiments were performed in 5 replicates. Confocal microscopy of uninfected corms (control) and corms inoculated with wild-type Fox R1 was done. To observe in planta EGFP signal in different layers of corms and root cells, images were scanned in multiple planes (Z stack) using a confocal microscope (Leica TCS SP8 AOBS), and 3D images of corms and roots were constructed using Leica Application Suite X (LAS X) software and ImageJ [42], respectively. 

### 2.11. Statistical Analysis

Calculation of Fox R1 load was conducted using 5 replicates. Results are expressed as mean ± standard deviation. Data were statistically analyzed by ANOVA using IBM SPSS statistics version 26 [43]. The Multiple Duncan multiple range test was performed for analyzing differences between mean values at significant levels (*p* < 0.05). 

## 3. Results

### 3.1. Molecular Characterization and Disease Assays of Fox R1

Five housekeeping genes, namely *EF-1α*, *Bt*, *H3*, *Act,* and *Cal* were used to characterize Fox R1 up to *forma specialis* level. The amplicon size obtained was 350 bp for *Bt*, 350 bp for *H3*, 500 bp for *Cal*, 300 bp for *Act,* and 700 bp for *EF-1α*. The sequences for these amplicons were submitted to GenBank under accession numbers *KJ866867–KJ866871* (Table 2). Based on the comparison of these sequences, Fox R1 was found to be closely related to f.sp. *ciceris* (*H3*), f.sp. *zingiberi* (*Cal*) and f.sp. *Cubense* (*Bt*). However, based on the *Act* gene and *EF- 1α* gene, Fox R1 was found to be closely related to *Fusarium oxysporum* strain FJDO-1 and *Fusarium oxysporum* isolates A010P, respectively (Table 2) (Figure S1). Further, the disease assays conducted on four *iridaceous* plants other than saffron, namely *Gladiolus*, *Sparaxis*, *Tritonia,* and *Ixia,* revealed that Fox R1 infects *Sparaxis* and *Tritonia* but no infection was observed in *Gladiolus* and *Ixia*. In Saffron, *Sparaxis,* and *Tritonia* microcopy and ITS phylogeny results revealed that the infection in these plants was caused by Fox R1 only (Table 3).

### 3.2. Route of Infection of Fox R1 in Saffron

**Figure 3:**
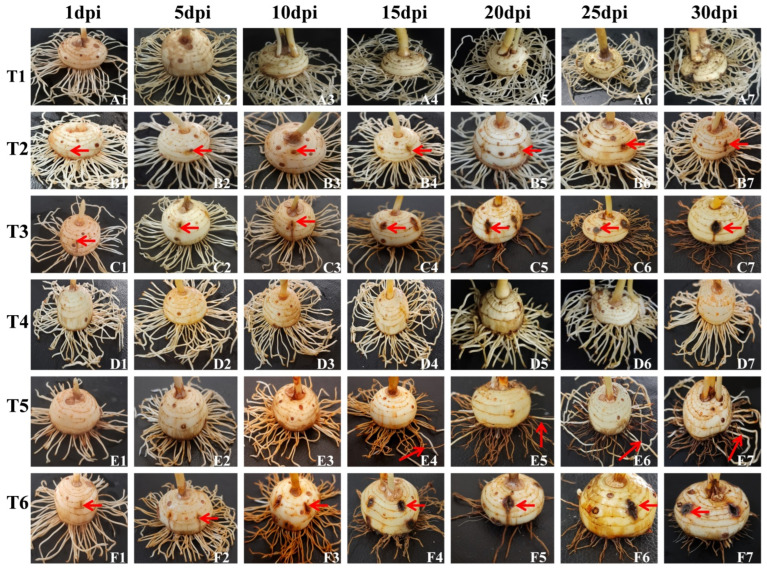
Corm images with different treatments at 1, 5, 10, 15, 20, 25, and 30 dpi (days post inoculation). Six different treatments were given to corms. T1—no injury was given but *Fusarium oxysporum* R1 (Fox R1) spore suspension was added to soil, T2—only corm injury was given and inoculated with sterile distilled water, T3—corm injury was given and inoculated with Fox R1 spore suspension, T4—only root injury was given and inoculated with sterile water, T5—only root injury was given and inoculated with Fox R1. Arrows in T5 indicate that the uninjured roots were free of infection and were grown normally. T6—the injury was given to both roots and corms and inoculated with Fox R1. In T2, T3 and T6 arrows indicate the injury points on the corm surface.

**Figure 4:**
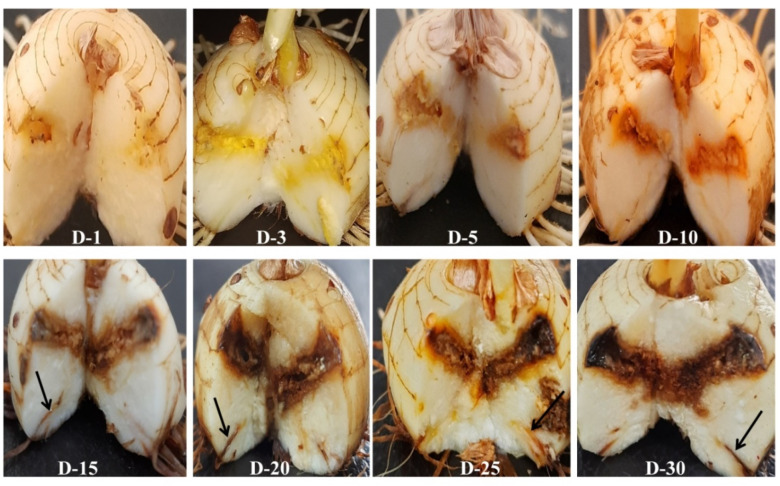
Symptoms of corm rot caused by Fox R1 infection in the dissected corms of T3 (corm injury + Fox R1) treatment. D1–D30 represents days post-inoculation. Arrows indicate the growth of Fox R1 in the root primordia.

**Figure 5:**
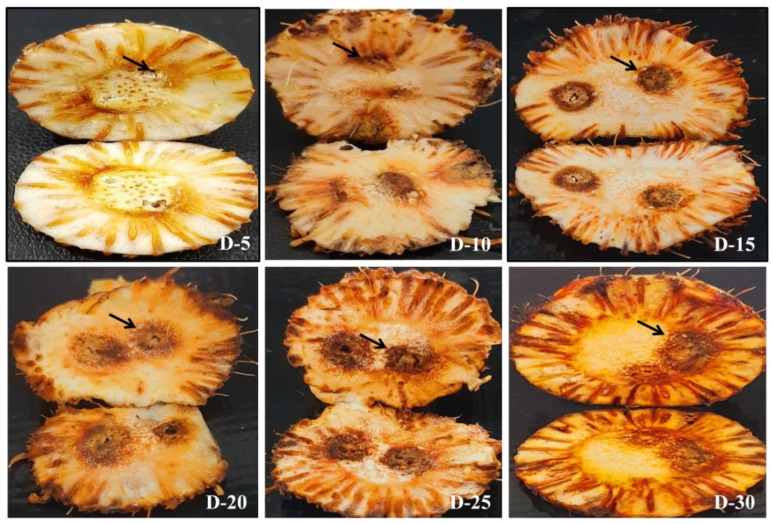
Symptoms of corm rot in the corm basal plate of T3 (corm injury + Fox R1) treatment. D-5, D-10, D-15, D-20, D-25, D-30 represent days post inoculation. Arrows indicate the injury point from where infection spread all over the basal plate.

Corms with different treatments as described in Figure 2 were monitored for disease progression up to 30 days post-inoculation (dpi). No symptoms were observed in roots and corms in T1 up to 30 dpi (Figure 3A1–A7). In T2 (CI only), there was a slight brown spot around the injury point at 5 and 10 dpi (Figure 3B2,B3) and increased browning at 15, 20, 25, and 30 dpi (Figure 3B4–B7). In T3 (CI + Fox R1), symptoms of corm rot increased from 1 to 30 dpi. The intensity of symptoms ranged from pale yellow around the injury point at 1, 5 dpi to enhanced browning at 10, 15, and 20 dpi followed by blackening around the injury point at 25, 30 dpi on the surface of the corms (Figure 3C1–C7). Additionally, similar symptoms were observed inside the dissected corms in T3 from 1–30 dpi (Figure 4). Visible symptoms were also observed at the corm basal plate from 5 dpi to 30 dpi (Figure 5). Figure 3C4–C7 clearly shows the spreading of infection from the corm basal plate to lateral roots. At 15 dpi, half of the roots showed browning due to the presence of Fox R1, later on, all the roots were completely infected and turned dark brown in color (Figure 3C4–C7). 

**Figure 6:**
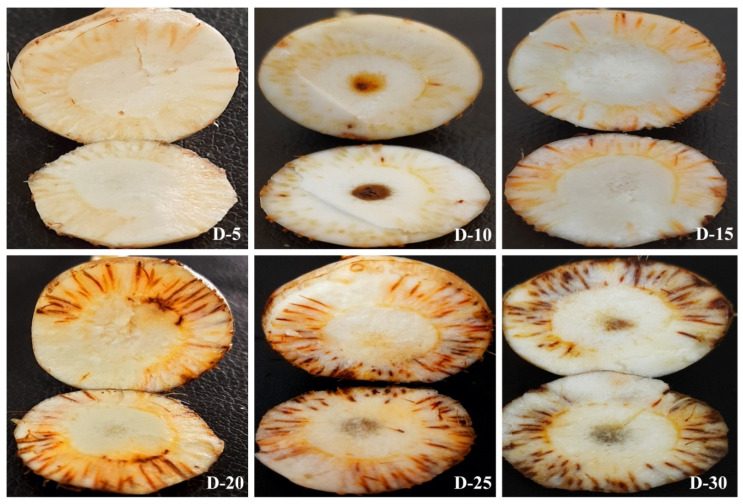
Symptoms of corm rot in the corm basal plate of T5 (root injury + Fox R1) treatment indicated the movement of Fox R1 from roots to the corm basal plate as no injury was given to corms.

In, T4 (RI only), a slight brown spot at the root tips was observed at 10, 15, 20, 25, and 30 dpi (Figure 3D1–D7. In T5, (RI + Fox R1), browning of root tips started at 1 dpi and then with increasing multiplication of pathogen inside the roots, symptoms became more severe (Figure 3E1–E7). Interestingly, in T5, only the injured roots become infected, but uncut (uninjured) roots showed normal growth (indicated by arrow) and were white as compared to injured roots on the same corm. Further, to track the movement of Fox R1 from roots to corm, the basal plate of corm from where roots were emerging was excised and observed. Visible symptoms were observed at 15, 20, 25, and 30 dpi with increasing intensity (Figure 6). Based on the results an inference was drawn, that injury is required for the entry of Fox R1 as the mere presence of Fox R1 did not cause infection to corm and roots up to 30 dpi. Additionally, in T5 uncut roots were uninfected and showed normal growth when compared to the cut roots.

In T6, wherein both roots and corms were injured (RCI + Fox R1) symptoms were observed both on corm and roots and intensity kept on increasing from 1 to 30 dpi, additionally at 25 and 30 dpi, only a few of the roots remained attached to the corms (Figure 3F1–F7). Among all the treatments in presence of Fox R1, injury to both roots and corm (T6) is the severest case followed by corm injury only (T3) and then root injury (T5). 

### 3.3. Confirmation of Infection of Fox R1 by Semi-Quantitative PCR and q-PCR

#### 3.3.1. Semi-Quantitative PCR

Amplification of the 100 bp region of ITS gene from the genomic DNA of infected tissue confirmed the presence of Fox R1. There was no cross-amplification of the ITS gene from the saffron plant genome, as primers specific to Fox R1 were designed. In T1, no amplification was seen in any of the samples, which confirmed the absence of Fox R1. In T2 and T4, also no amplification was observed. In T3, (CI + Fox R1), amplification of 100 bp of ITS gene confirmed the presence of Fox R1 in the corms (Figure S2a). However, amplification in the 100 bp region was also observed in the root samples of the same corm at 10, 15, 20, 25, and 30 dpi (Figure S2b). The presence of symptoms and amplification of the ITS gene in roots confirmed the movement of Fox R1 from corm to roots as no injury was given to roots in treatment T3 (CI + Fox R1). Similarly, in T5 (RI + Fox R1), amplification was seen from 1–30 dpi (Figure S3a). Further, amplification at 15, 20, 25, and 30 dpi was observed from the DNA sample isolated from the corm basal plate (Figure S3b). This amplification indicated the transfer of Fox R1 from infected roots to the corm basal plate as no injury was given to corms. 

#### 3.3.2. Standard Curve Analysis and Quantification of Fox R1 Load by q-PCR

**Figure 7:**
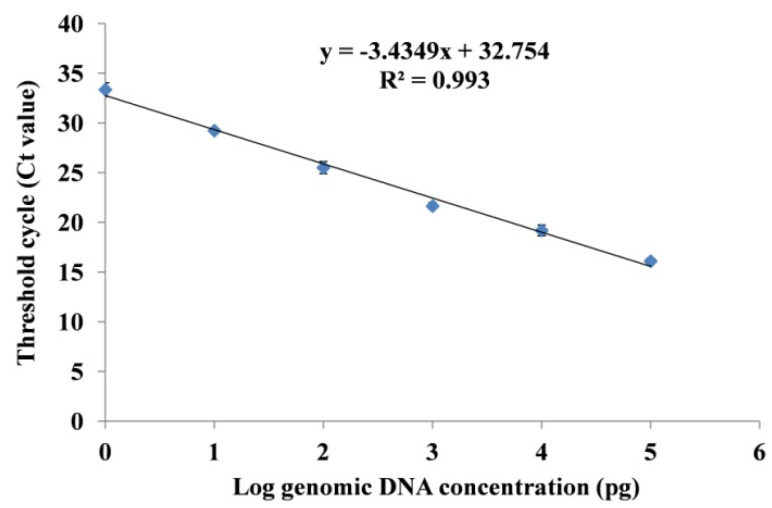
Standard curve of 10-fold serially diluted DNA of Fox R1 (100 ng−1 fg) for the quantification of Fox R1 in infected roots and corms.

To quantify the Fox R1 load in infected roots and corms, a standard curve from known DNA concentration was prepared. The standard curve showed the linear relationship between the Ct values and log genomic DNA concentration (Figure 7). The regression equation determined was Y= −3.4349x + 32.754 (R² = 0.993, E = 95.49%) with a detection limit of 1 pg of DNA concentration (Ct = 33.5 ± 0.69). The Ct value of 34 was used as the cutoff limit for the detection of Fox R1 [44]. 

The load of Fox R1 in infected corm and roots was quantified at 1, 3, 5, 10, 15, 20, 25, and 30 dpi in inoculated saffron corm and roots. In mock-inoculated samples no amplification was seen. In T3 (CI + Fox R1) the quantity of Fox R1 DNA in corm ranged from 11.77 ± 1.9 pg/μL at 1 dpi to 10,723.09 ± 222.1 pg/μL at 30 dpi. (Table 4). In roots of the same treatment quantity of Fox R1 DNA ranged from 3.11 ± 0.9 pg/μL at 5 dpi to 1489.97 ± 169.7 pg/μL at 30 dpi. This further confirmed the downward movement of Fox R1 from corm to roots that occurred at 5 dpi. In T5, (RI + Fox R1), the quantity of Fox R1 DNA in roots ranged from 11.01 ± 2.5 pg/μL at 1 dpi to 1561.167 ± 176.4 pg/μL at 30 dpi and in the corm basal plate of the same treatment, the quantity of Fox R1 DNA ranged from 3.26 ± 1.2 pg/μL at 1 dpi to 3076.45 ± 167.3 pg/μL at 30 dpi. This confirmed the upward movement of Fox R1 from roots to corm basal plate that occurred at 10 dpi. Further, the results of semi-quantitative PCR and q-PCR were compared and tabulated in Table 4. 

### 3.4. Determinations of Fox R1 Load at the Site of Infection 

The load at different sites was determined by CFU calculation and represented as the number of Fox R1 colonies/ gram of tissue. No colonies were observed in T1, T2, and T4. However, in T3, T5, and T6 colonies were observed, and the maximum load was at 30 dpi in both corms and roots, respectively. There was a significant difference in Fox R1 concentration at various sites on different days of each treatment. For easy comprehension, the results have been summarized in Table 5. The colonies grown were randomly observed under the light microscope and characteristics sickle shape spores of *Fusarium oxysporum* R1 were observed. Additionally, amplification of the 100 bp region of the ITS gene confirmed that the colonies were of Fox R1. 

### 3.5. Tagging and Expression of EGFP in Transformed Fox R1 at Different Time Points 

**Figure 8:**
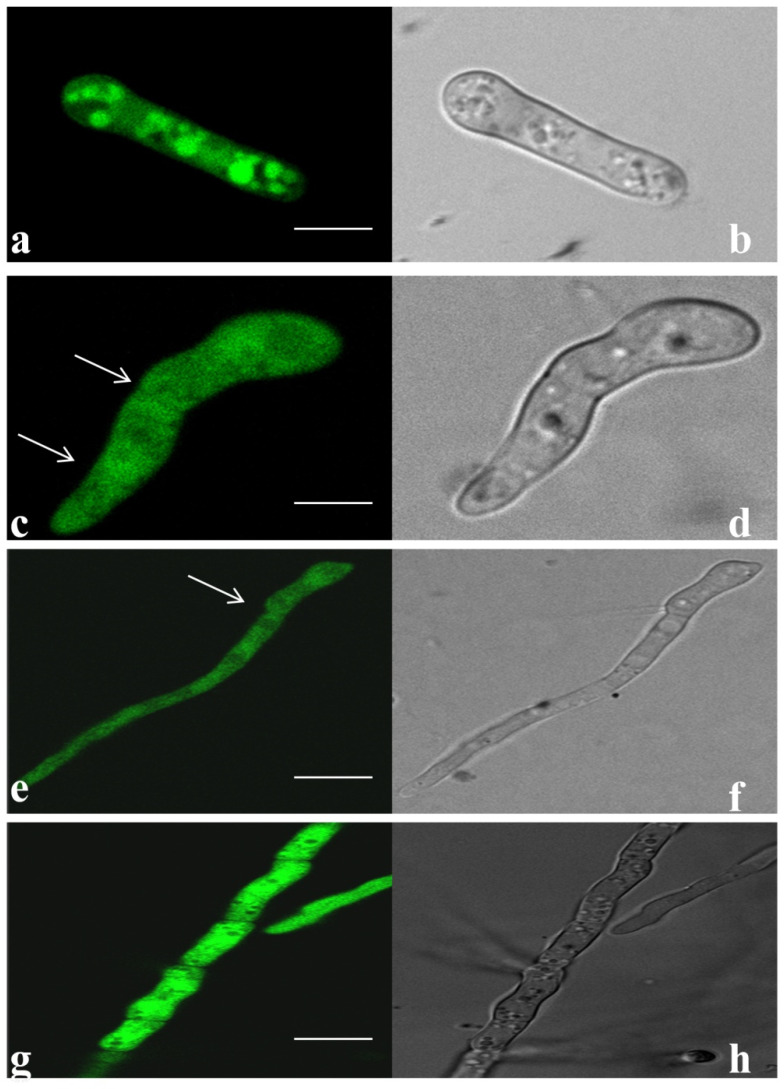
Expression of EGFP in transformed Fox R1. (**a**,**b**); Microconidia, (**c**,**d**); macroconidia, (**e**,**f**); germinating macroconidia, (**g**,**h**); Fox R1 hyphae. Each bar represents 10 µm. (**b**,**d**,**f**,**h**) are the transmitted light images. Arrow indicates the septation in macrospores.

Successful transformation of Fox R1 was performed with *Agrobacterium tumefaciens* strain-GV3101 carrying pBIF-EGFP vector after co-cultivation with Fox R1 spores (1 × 10^6^ spores/mL) in presence of acetosyringone (0.2 mM), leading to hygromycin resistance at 100 µg/mL concentration. Approximately 4–5 transformants/10^6^ spores/Petri plate (90 mm), were obtained. The expression of EGFP in the transformed Fox R1 was confirmed by observing the harvested spores under the confocal fluorescence microscope. Uniform fluorescence was observed in all the transformants, and no auto-fluorescence was detected in the wild-type Fox R1. In addition, to establish that the EGFP has integrated stably into the genome, conidia were studied at different time intervals on a glass slide, to confirm the transfer of genes in various generations. Confocal microscopy of Fox R1 spores indicated the expression of the pBIF-EGFP vector at different developmental stages, i.e., microconidia, macroconidia, and fungal hyphae as shown in Figure 8.

### 3.6. Conformation of Transformants

Genomic PCR amplification of the 720 bp region of the *egfp* gene confirmed the presence of T-DNA in transformants (Figure S4a). Southern hybridization of the randomly selected six isolates of Fox R1-EGFP transformants along with the wild type resulted in the different band sizes and indicated random integration of the reporter genes. In most of the transformants, single-copy integration of the gene was observed, while multi-copy integrations were also observed in a few transformants (Figure S4b).

### 3.7. Invasion and Colonization of Fox R1 

**Figure 9:**
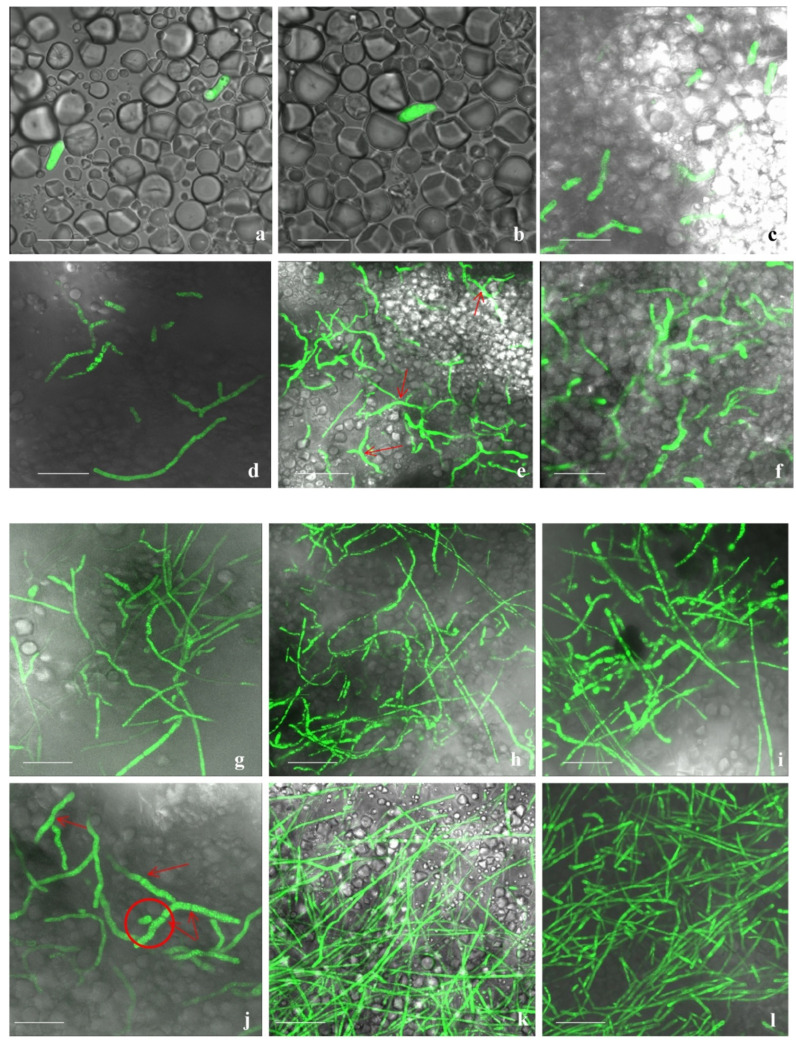

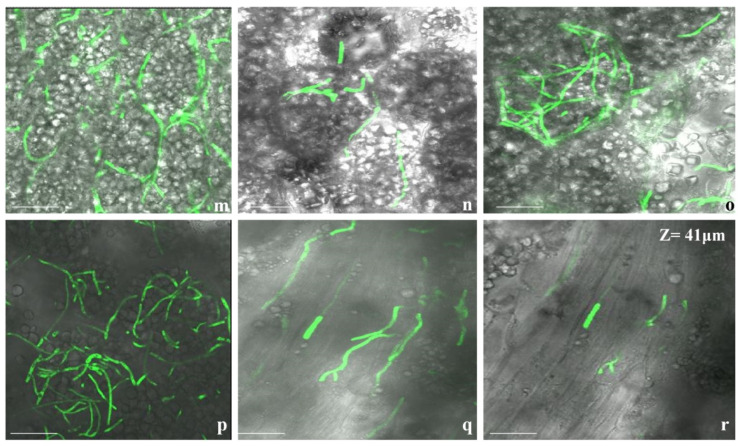
Different stages of colonization of tagged Fox R1 in infected corm tissue of saffron. (**a**,**b**); Corm tissue at 6 hpi, characteristics sickle shape spores clearly visible, (**c**,**d**); spore germination at 12 and 18 hpi, (**e**); 1 dpi, Y shape structure were seen (indicated by arrows), (**f**–**h**); hyphae continue to grow and covered the tissue surface, at 2 dpi it started penetrating inside the inner layers of the tissue, (**i**,**j**); septate hyphae clearly visible (indicated by arrows) at 5 dpi, (**k**,**l**); hyphae at 8 and 12 dpi few of the hyphae were seen inside the corm tissue, (**m**,**n**); represents the Fox R1 in the extracellular spaces of the corm tissue, (**o**,**p**); represents the Fox R1 inside the corm cell at 20 dpi and (**q**,**r**); represents the Fox R1 in the vascular tissue. (**a**,**r**); represents merged images after z- stacking using laser scanning confocal microscope. Each bar represents 10 µm.

In the disease assay of saffron corms and roots, using EGFP-transformed Fox R1 the infected tissues showed increasing rot symptoms with an increase in days post inoculation (dpi) in pots. In uninfected corm tissues, cells were visible with starch granules inside the cells (Figure S4a,b). During infection caused by Fox R1 the tissue at the site of infection became spongy and as sections were cut manually so in the infected tissues intact cells were not visible as compared to the uninfected corms. Microscopy of corm tissue infected with wild-type Fox R1 was also completed and hyphae were observed under bright fields only (Figure S4c,d). Confocal microscopy of the infected corms with tagged Fox R1 was performed starting from 6 h to 5, 8, 12, and 20 dpi. At 6 h post-inoculation, a few of the sickle shape spores (microconidia) were observed in corm tissues between the starch granules (Figure 9a,b).

**Figure 10:**
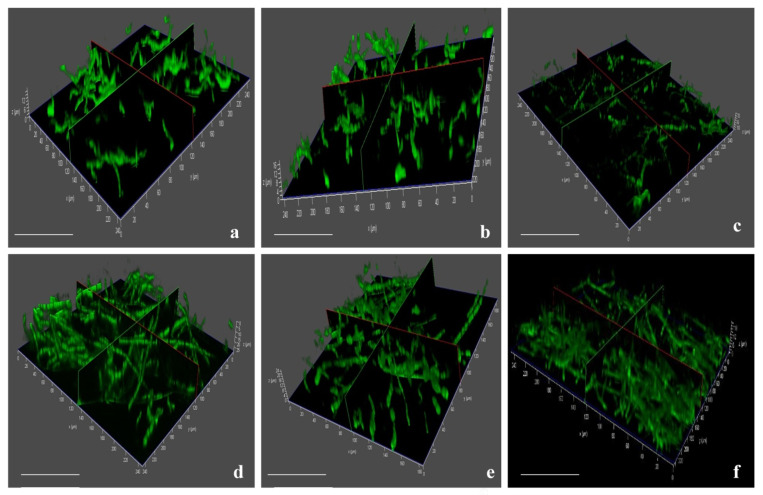
(**a**–**f**) Represents the three-dimensional (3D) images of corm tissue at 1, 2, 3, 4, 5, and 12 dpi, respectively. Z-axis represents the thickness of the infected tissue. Mycelia were present in all the layers of the tissue (from the base layer to the upper surface). Each bar represents 10 µm.

**Figure 11:**
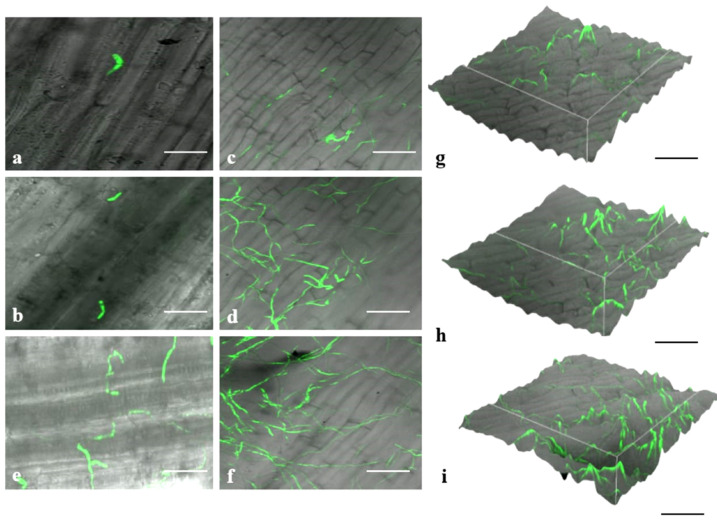
Different stages of colonization of tagged Fox R1 in root cells of saffron. (**a**); Germinating spores at 12 hpi, (**b**); germinating spores entering the vascular tissue, (**c**); Fox R1 hyphae start colonizing the root surface and hyphae attached themselves to root surface along the junction of epidermal cells at 1 dpi, (**d**); 5 dpi mycelia covered the root surface and formed mesh of hyphae, (**f**); at 10 dpi, elongated hyphae were observed, (**e**); hyphae entering the xylem tissue, (**g**,**h**,**i**); are the corresponding 3-D images of infected roots at 1,5,10 dpi respectively Each bar represents 10 µm.

Spore germination starts at 12 hpi (hours post-inoculation) (Figure 9c) and continues to germinate till 18 hpi (Figure 9d). At 1 dpi, most of the germinating conidia formed Y shape hyphae (Figure 9e). From 2 to 3 dpi, hyphae continued to grow and started to penetrate the corm tissues (Figure 9f,g). At 4 dpi, elongated hyphae were observed that extend from one end to the other ends of the tissue and formed a mesh of hyphae (Figure 9h). Septate hyphae for the first time were observed at 5 dpi (Figure 9i,j). Fox R1 showed continuous growth from 5 dpi to 8 dpi, at 8 dpi dense mesh of hyphae was observed and at 12 dpi Fox R1 completely covered the surface of the tissue (Figure 9k,l). Up to 12 dpi, Fox R1 remained in the extracellular spaces of the corm tissue (Figure 9m), and surprisingly, in the later stages, Fox R1 entered the corm cells and grew inside the cells as observed at 20 dpi (Figure 9n–p). Fox R1 was also observed in the xylem vessels of corm tissues (Figure 9q,r). Figure 10a–f, represents three-dimensional (3D) views of Fox R1 in the tissue at different dpi, and Fox R1 was found to be present in different layers of the tissue. Microscopic examination of roots depicted germinating spores after 12 h post-inoculation (Figure 11a) some of the germinating spores entered the xylem vessels as well (Figure 11b). At 1 dpi, hyphae started to colonize the root surface (Figure 11c) and at 5 dpi root surface was covered with dense mycelium that formed mesh (Figure 11d). At 10 dpi elongated hyphae were observed (Figure 11f). At 8 dpi, Fox R1 was observed growing inside the xylem vessels (Figure 11e). Figure 11 g–i are the corresponding 3D images of the roots at 1, 5, and 10 dpi, respectively.

## 4. Discussion

*F*. *oxysporum* species complex (FOSC) is one of the most important groups belonging to the *Fusarium* plant pathogens. Strains of FOSC are known to cause wilts and rots in more than 100 agronomical important crops such as tomato, banana, chickpea, watermelon, pea, sweet potato, legumes, etc. [45,46,47,48,49]. *Fusarium oxysporum* is among the first five most devastating plant pathogens, with about 150 formae speciales [50,51,52]. The forma specialis *gladioli* has been reported to infect the members of *Iridaceae* family and is also reported to be a major pathogen of *Crocus sativus*, the world over [14,15,20,21]. Gupta and coworkers in 2011 classified the saffron corm rot-causing *Fusarium oxysporum* isolated from Kishtwar, in India as *Fusarium oxysporum* f.sp. *gladioli* [15]. However, in our previous study, we found the pathogenic *Fusarium oxysporum* isolated from Kashmir was different from *Fusarium oxysporum* f.sp. *gladioli* based on ITS sequence identity but was found similar to *Fusarium oxysporum* f.sp. *dianthi* [16]. The taxonomy of *Fusarium* is controversial due to the lack of a universal species concept [53]. For species-level identification, DNA-based markers such as ITS region and other housekeeping genes are standards for the diagnosis of fungal pathogens. In continuation of our previous observations, in the present study, multiple phylogenetic markers were used to characterize Fox R1. Based on the molecular phylogeny of selected housekeeping genes mentioned in Section 3, Fox R1 was found to be closely related to *Fusarium oxysporum* f.sp *ciceris*, f.sp *zingiberi,* and f.sp *cubense.* These have been reported as pathogens of chickpeas, ginger, and banana, respectively [54,55]. However, gene sequence-based phylogenies are not sufficient to determine the host specificity of the Fox strains [56]. Since Fox R1 could not be characterized unambiguously by molecular phylogenetic analysis alone; disease assays were performed to classify Fox R1 up to *forma specialis* level. So far, Fox f.sp. *gladioli* are the most commonly reported Fox pathogen of saffron which indicates that *Gladiolus* is also a host. However, Fox R1 did not infect *Gladiolus* in the present study. Therefore, it is different from *F*. *oxysporum* f.sp. *gladioli,* already reported corm rot agent of saffron.

Previously, all the *F. oxysporum* species causing disease in iridaceous crops were generally accepted as f.sp. *gladioli*, but Roebroeck in 2000 based on the pathogenicity tests confirmed the presence of 10 different pathotypes of *Fusarium oxysporum* [56]. On the basis of phylogenetic analysis and host specificity, Fox R1 was classified as *F*. *oxysporum* f.sp. *iridiacearum* race 4, as all the three infected genera in the present study, belonged to the family *Iridiaceae*. Correct identification of the pathogen is the first step toward its diagnostics and subsequent disease management. Fox R1 has been identified up to *forma specialis* in the present study and the tools developed are available for further evolution of its diagnostic kits. 

There are plenty of reports on the mode and mechanism of infection by *Fusarium oxysporum* on roots of various plants [39,49,57,58,59], but no reports are available on mode and site of *Fusarium oxysporum* infection and disease progression in corms except in rhizome of banana corms [59,60]. Since there is no such study on the saffron *Fusarium* pathosystem, so to decipher the route and mode of action, Fox R1 was used to infect both roots and corms individually and simultaneously. To confirm the presence of Fox R1 and to track its movement semi-quantitative PCR and q-PCR using *Fusarium oxysporum*-specific ITS gene primers were performed and its load in the infected tissue was also determined by CFU/g of infected tissue. Similar to the present study, Vasilescu and Blanchard in 2002 have used a semi-quantitative PCR technique for the detection of *Alternaria brassiccola* and *Alternaria japonica* in infected seeds of Crucifers [61]. Xiao and coworkers in 2013 reported the use of FOF1/FOF2 primers for the identification of *Fusarium* species in infected roots, rhizome, and pseudostem of the banana plant. The researchers further used Foc1/Foc2 primers to confirm the species as Foc4, using semi-quantitative PCR [62]. 

q-PCR is considered the gold standard for the detection and quantification of specific DNA sequences from any sample [63]. This technique is commonly used for the detection and quantification of soil-borne fungal pathogens causing diseases in plants [64]. In the present study, q-PCR was found to be 10 times more sensitive than the semi-quantitative PCR. The technique of q-PCR has been widely used for the quantification of *Fusarium oxysporum* in plant tissues such as the quantification of *Fusarium oxysporum* f.sp. *ciceri* in chickpea [65], *Fusarium oxysporum* f.sp. *cubense* in banana [66]. Similar to the present study, Singh and Kapoor in 2018have used the technique of semi-quantitative PCR and q-PCR for the rapid detection of *Fusarium oxysporum* f.sp. *carthami* in safflower tissues [44]. 

In the present study, Fox R1 moved bidirectionally from corms to roots and from roots to corms. In T3 (CI + FoxR1) roots get infected, become dark brown in color, and withered after 30 dpi. Since in this case no infection was given to roots, results indicated movement of Fox R1 from corm to roots (Figure 3C4–C7). This was further confirmed by semi-quantitative PCR, q-PCR, and CFU count. The maximum load was at 30 dpi in the corm basal plate and roots at 30 dpi (Table 4 and Table 5). However, q-PCR was able to quantify Fox R1 in roots of T3 at 5 dpi whereas; semi-quantitative PCR and CFU determined the load at 10 dpi. This can be attributed to the high sensitivity of q-PCR assays compared to semi-quantitative PCR.

Similarly, in T5 (RI + Fox R1) the basal plate of the corm was also infected in addition to the roots (Figure 6) but uninjured/uncut roots were growing normally without any symptoms of infection (Figure 3E1–E7) as indicated by arrows. The dark browning of the infected tissues is due to the degradation of phenol in presence of the enzyme polyphenol oxidase secreted by the pathogen at the site of infection [67,68]. Injury is important for infection in saffron and the same has also been reported in plants such as tomato [69], melon [70], banana [49] and grass pea [71]. In saffron corms (under natural conditions) the injury is caused during the digging of corms, storage, handling and transportation [7]. Additionally, parasitic nematodes and rodents have also been reported to enhance the event of infection by causing wounds, thereby resulting in the release of exudates and attracting microbial pathogens [72]. In this preliminary study, the recommendation being made is that care should be taken to avoid injury to corms during storage and sowing of corms. 

In saffron plants, two types of roots are present fibrous roots and contractile roots [73]. The fibrous (main) roots emerge from the primordial at the inner edge of the cortex of the corm and cover the base of the mother corm during its vegetative phase. The fibrous roots of the saffron plant are the main absorbing roots that provide nutrition to the plant. Fox R1 enters the fibrous roots through injury or wounds and multiplies inside the roots. Afterward, the pathogen invades the vascular bundles and reaches the basal plate of the corm (cortex cells) through primordia after 10 dpi as depicted by q-PCR. Up to 30 dpi, Fox R1 movement was restricted to cortex cells only and no visible symptoms appeared on the surface of the corm. The intensity of symptoms and load at the basal plate increased to 30 dpi (Figure 6, Table 4 and Table 5). However, its movement from corm to roots (downward) was based on visible symptoms on roots. Fox R1 traveled from corm through root primordia and invaded the roots. The movement of Fox R1 from corm to roots has been shown by the rot symptoms of the primordia and further confirmed by Fox R1 CFU count and semi-quantitative PCR (Figure S2b, Table 4 and Table 5). 

Similar to the present study, Xiao and coworkers in 2013 reported the upward movement of gfp-tagged *Fusarium oxysporum* f.sp. *cubense* race 4 from roots to rhizome then to the pseudostem. Foc4 first penetrated the young roots by forming conidia or germ tubes. The hyphae reached the xylem of the rhizome and pseudostem at 17 dpi and plants died at 24 dpi [62]. In 2017, Li and coworkers also studied the colonization of two races of *Fusarium oxysporum* f.sp. *cubense* race 1 (Foc1) and race 4 (Foc4) and found that after colonization in roots through root hairs and epidermis, Foc4 showed upward movement into the rhizome (corm) but Foc1 was not found in the rhizome (corm) up to 2 months [49]. The movement of gfp-tagged *Fusarium oxysporum* f.sp. *cubense* in the susceptible cultivar of banana was studied and was found that the pathogen moved from the initial site of infection, i.e., roots (uninjured in contrast to saffron where an injury is required), through the rhizome (corm), into the pseudostem and finally to the leaves, at the top [60]. In 2018, Zhang and coworkers monitored the infection pattern of *Fusarium oxysporum* f.sp. *cubense* race 4 in *Musa acuminata* Pahang (resistant) a highly susceptible Brazilian cultivar. In both accessions, Foc TR4 invaded the roots through the wound (similar to saffron) and reached vascular bundles at 3 dpi and corms at 7dpi [59]. However, in the present study, Fox R1 reached corms at 10 dpi from the infected roots. There is no report available on the downward movement of the pathogen from (corm/rhizome) to roots as observed in the present study. The experiment in the present study was performed at the start of the flowering phase of the saffron life cycle till 30 days to investigate the movement of Fox R1 towards the aerial parts of the plant, to obtain a comprehensive picture it needs to be studied for at least one complete life cycle of the corm.

Further, *Fusarium oxysporum* R1 was tagged with a green fluorescent protein to track its movement inside the roots and corms and to study its growth pattern. After transformation, the transformants obtained had stable integration of fluorescent genes as evident by the expression of green fluorescent protein in different developmental stages under a confocal microscope at various time points and by southern hybridization. High-intensity fluorescence was observed in conidia and hyphae of transformed Fox R1. The expression of the fluorescent protein was observed in microconidia (2 dpi), germinating macroconidia (4 dpi), and fungal hyphae (6 dpi) (Figure 8). Similarly, expression of fluorescent protein DsRed-Express was visualized in microconidia, macroconidia, and chlamydospore of *Fusarium oxysporum* f.sp. *ciceri*, a pathogen of chickpea [39], expression of GFP protein in conidia and hyphae of *Fusarium oxysporum* f. sp. *cubense*, *a* pathogen of banana [62], *Fusarium oxysporum* f.sp. niveum, a pathogen of watermelon [74], *Fusarium verticilliodies*, a pathogen of wheat [75], *Fusarium oxysporum* f. sp. *cubense* tropical race 4, a pathogen of *Musa acuminata* pahnag [59] and *Fusarium oxysporum,* a pathogen of *Echeveria* plant [76]. Characteristic sickle shaped spores (microconidia) at 6 h and germinating spores at 12 h were seen in tagged Fox R1 (Figure 9a–c). Fox R1 hyphae formed a network on the surface and inside of the corm tissue at 2 dpi which kept on increasing. Septate hyphae were observed at 5 dpi in the corms (Figure 9h). At 8, 12 dpi denser mesh was observed. Fox R1 up to 12 dpi remains extracellular as seen in a single section of cells (Figure 9m), where Fox R1 was found localized around the cell surfaces of the tissue. After that, Fox R1 penetrates inside the cell (Figure 9n) and grows inside, suggesting the intracellular localization of Fox R1 in the later stages of infection (Figure 9o–p). The uninjured corms and roots did not show any fluorescence even though the soil was inoculated with tagged Fox R1 but hyphae were observed only in the injured corms inoculated with wild-type Fox R1 (Figure S4c,d). In 3D imaging, Fox R1 showed its presence in multiple planes of the corm tissue (Figure 10). There are very few reports available in the literature on the colonization of Fox in corm tissues; however, [60,62] have reported the colonization of gfp-tagged *Fusarium oxysporum* f. sp. *cubense* in the banana rhizome (corm) that travels upwards from the roots. Xiao and co-workers in 2013 reported very few fungal hyphae in the rhizome of the banana plant, the Foc first enters the plant through roots and moves upwards to the rhizome and then to the pseudostem. The highest density of Foc was in found in the pseudostem, i.e., 10.4 × 10^2^ CFU/gm of tissue, followed by 1.02 × 10^2^ CFU/gm of tissue in the roots and only 0.24 × 10^2^ CFU/gm of tissue in the rhizome [62]. 

Germinating Fox R1 spores were observed in roots at 12 h and also some of them had entered the xylem vessels (Figure 11a,b). Subsequently, the hyphae found attached to the cell surface at 1 dpi and 5 dpi was observed progressing through extracellular spaces forming a mesh-like network. Hyphae reached xylem tissue at 8 dpi (Figure 11e). At 10 dpi when almost all of the roots were infected (as evident from root rot) a large number of conidia germinating from the conidiogenous cell along with hyphae were observed (Figure 11f). Further, in 3D images of the roots, Fox R1 was found in the different layers of the roots. Similar to the present study, Zhang and co-workers in 2015 studied the colonization pattern of gfp-tagged *Fusarium oxysporum* f.sp. *niveum* causing *Fusarium* wilt in watermelon at different time intervals [74]. Fox f.sp. *niveum* was able to colonize roots but did not require wounding of roots for its entry, unlike saffron. At 5 and 6 dpi, mycelia entered the xylem vessels and grew there. In contrast to the present study, Warman and Aitken in 2018 have reported the presence of Foc in the roots of Cavendish banana at 20 dpi and hyphae network at 30 dpi but Foc followed the same pattern for progression as observed in the present study in saffron [60]. Recently, Sampaio et al. in 2021 have found that at 7 dpi *Fusarium oxysporum* f.sp. *pisi* completely colonizes the roots of susceptible pea accessions and forms a dense network [71]. In bananas, the infection occurs through secondary and tertiary roots, primary (large) roots rarely get infected directly. Rhizome and pseudostem, if infected, the infection remains localized to these parts only. So, roots are the major entry points of the pathogens in bananas [26].

## 5. Conclusions

In the present study, the classification of strain R1 was performed based on housekeeping genes and disease assays and it was classified as *Fusarium oxysporum* R1 f.sp. *iridacearum* race 4. Further, it was found that in saffron both roots and corms serve as the sites for the entry of Fox R1 and it travels from corm to roots and vice versa. In addition, by real-time imaging and fungal load estimation and quantification, it was clear that the case of saffron injury is required for the infection of Fox R1 and it needs to be further verified for other pathogens associated with corm rot disease. Moreover, the q-PCR method developed can be used for the quantification of Fox R1 in infected soils as well as saffron plants. This is the first report on the molecular understanding of the saffron Fox R1 pathosystem and can prove useful in the detection and management of other corm rot causing agents in saffron. 

## Figures and Tables

**Table 1:** Experimental layout for checking the *Fusarium oxysporum* R1 load in the infected saffron plant.

S. No	Treatments	Description of Treatment		Site for DNA Isolation
		Injury	Fox R1	
1	T1	No	−	Root and corm
2	T2	Corm injury	−	Corm injury point
3	T3	Corm injury	+	Corm basal plate and roots
4	T4	Root injury	−	Root tips
5	T5	Root injury	+	Roots and corm basal plate
6	T6	Root + corm injury	+	Corm injury site and roots

T1—No injury + Fox R1, T2 (CI − Fox R1), T3 (CI + Fox R1), T4 (RI − Fox R1), T5 (RI + Fox R1), T6 (RCI + Fox R1). CI—Corm injury. RI—Root injury. RCI—Root corm injury.

**Table 2:** List of housekeeping genes used in the present study for the identification of *Fusarium oxysporum* R1.

S.No	Housekeeping Genes	Amplicon Size (bp)	GenBank Accession Number	Closest Neighbor Based on Phylogeny	Identity (%)	Accession Number
1	Actin	300	*KJ866867*	*Fusarium oxysporum* strain FJDO-1	99.55%	*MK895954*
2	Translation elongation factor 1α	700	*KJ866868*	*Fusarium oxysporum* isolate A010P	100%	*MN191811*
3	β-tubulin	350	*KJ866869*	*Fusarium oxysporum* f.sp. *cubense* isolate Foc37	100%	*MF668108*
4	Calmudlin	500	*KJ866870*	*Fusarium oxysporum* f.sp. *zingiberi* isolate Gf-VA-3	99.05%	*MT802441*
5	Histone 3	350	*KJ866871*	*Fusarium oxysporum* f.sp. *ciceri*	980.5%	*AF346506*

**Table 3:** Disease assays of Fox R1 were conducted on four other members of the family *Iridaceae*.

Genus	Symptoms	Microscopy	ITS Phylogeny	Infection by Fox R1
*Crocus*	+	Macrospores (1–3 septa), curved spores.	Fox R1	+
*Gladiolus*	−	Aseptate spores		−
*Ixia*	−	Single septate curved spores	-	−
*Sparaxis*	+	Macrospores (1–3 septa), curved spores.	Fox R1	+
*Tritonia*	+	Macrospores (1–3 septa), curved spores.	Fox R1	+

**Table 4:** Quantification of Fox R1 in infected roots and corms at different days post inoculation using Fox R1-specific primers.

		T3 (Injury to Corm Only + Fox R1)								T5 (Injury to Roots Only + Fox R1)							
S.No.		Corm				Roots				Roots				Corm Basal Plate			
	dpi	Symptoms (A)	Semi-Quantitative (B)	q-PCR (C)	Concentration (pg) (D)	Symptoms (A)	Semi-Quantitative(B)	q-PCR (C)	Concentration (pg) (D)	Symptoms (A)	Semi-Quantitative (B)	q-PCR (C)	Concentration (pg) (D)	Symptoms (A)	Semi-Quantitative (B)	q-PCR (C)	Conentration (pg) (D)
1	1	+	+	+/29.08 ± 0.91	+/11.77 ± 1.9	-	-	-	-	+	+	29.18 ± 0.8	+/11.01 ± 2.5	-	-	-	
2	3	+	+	+/27.13 ± 0.67	+/43.61 ± 5.62	-	-	-	-	+	+	28.15 ± 0.18	+/21.99 ± 4.8	-	-	-	
3	5	+	+	+/25.26 ± 0.12	+/153.82 ± 15.90	-	-	+/31.06 ± 0.65	3.11 ± 0.9	+	+	26.34 ± 0.33	+/74.12 ± 8.7	-	-	-	
4	10	+	+	+/23.67 ± 0.55	+/445.04 ± 39.7	+	+	+/27.19 ± 0.43	41.89 ± 3.5	+	+	24.74 ± 0.6	+/216.9 9± 19.8	-	-	30.99 ± 0.87	+/3.26 ± 1.2
5	15	+	+	+/21.47 ± 0.91	+/1948.94 ± 180.7	+	+	+/25.57 ± 0.31	124.29 ± 7.9	+	+	23.49 ± 0.12	+/502.11 ± 34.6	+	+	27 ± 0.54	+/47.593 ± 8.9
6	20	+	+	+/19.69 ± 1.01	+/6437.98 ± 192.99	+	+	+/23.08 ± 0.50	661.31 ± 49.7	+	+	22.59 ± 0.79	+/918.9 ± 51.7	+	+	24.8 ± 0.44	+/208.425 ± 40.7
7	25	+	+	+/19.32 ± 0.79	+/8253.15 ± 204.7	+	+	+/22.13 ± 0.59	1251.35 ± 104.7	+	+	22.09 ± 0.55	+/1348.96 ± 1.2.9	+	+	21.87 ± 0.19	+/1489.97 ± 118.7
8	30	+	+	+/18.93 ± 0.34	+/10,723.09 ± 222.1	+	+	+/21.87 ± 0.34	1489.97 ± 169.7	+	+	21.8 ± 0.54	+/1561.167 ± 176.4	+	+	20.79 ± 0.52	+/3076.45 ± 167.3

dpi-days post inoculation. (A); (+) indicate symptoms visible to naked eyes, (-) indicate no visible symptoms, (B); (+) indicate amplicon size of 100 bp obtained, (-) indicate no amplification, (C); (+) indicated Ct value < 34, (-) indicated Ct > 34,(D); values are the concentration of Fox R1 (pg/μL) unknown samples determined from the standard curve.

**Table 5:** In planta quantification of *Fusarium oxysporum* R1 load in different treatments by CFU method.

S.No.	Days Post Inoculation(dpi)	*Fusarium oxysporum* R1 Load (10^5^ CFU/gm of Tissue) in Different Treatments						
		T3 (Corm Injury + FoxR1)			T6 (Corm + Root Injury +Fox R1)		T5 (Root Injury + Fox R1)	
	Sites	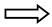	Corm basal plate	Roots	Corm tissue around injury point	Roots	Roots	Corm basal plate
1	1		0.16 ± 0.09 a	0	0.4 ± 0.10 a	0.15 ± 0.07 a	0.14 ± 0.08 a	0
2	3		0.42 ± 0.11 b	0	1.28 ± 0.08 b	0.5 ± 0.08 b	0.7 ± 0.06 b	0
3	5		1.50 ± 0.10 c	0	4.94 ± 0.11 c	1.42 ± 0.06 c	1.56 ± 0.12 c	0
4	10		5.78 ± 0.08 d	2.06 ± 0.08 a	9.6 ± 0.07 d	3.06 ± 0.11 d	3.52 ± 0.10 d	0
5	15		11.84 ± 0.11e	4.24 ± 0.11 b	13.2 ± 0.13 e	5.26 ± 0.05 e	5.2 ± 0.09 e	0.32 ± 0.13 a
6	20		15.50 ± 0.07 f	5.72 ± 0.06 c	17.19 ± 0.12 f	6.72 ± 0.13 f	6.68 ± 0.13 f	0.82 ± 0.08 b
7	25		18.12 ± 0.13 g	7.28 ± 0.07 d	20.92 ± 0.08 g	7.2 ± 0.12 g	7.22 ± 0.07 g	2.36 ± 0.05 c
8	30		21.32 ± 0.08 h	7.34 ± 0.27 e	24.9 ± 0.10 h	7.6 ± 0.03 h	7.61 ± 0.25 h	6.88 ± 0.09 d
9	*p*-value		0.00	0.00	0.00	0.00	0.00	0.00

Values are represented as (mean ± SD, *n* = 5). One-way ANOVA was performed at a significant level (*p* < 0.05). Means with different superscript letters (within the same row) indicate significant differences according to the multiple Duncan test (*p* < 0.05). Different letters within each column represent the significant difference.

## Data Availability

Not applicable.

## Supplementary Materials

The following supporting information can be downloaded at: https://www.mdpi.com/article/10.3390/jof8121246/s1, Figure S1A,B: Gel image showing the amplification of ITS gene of Fusarium oxysporum R1 in T4 (corm injury + Fox R1), Figure S2A,B: Gel image showing the amplification of ITS gene of Fusarium oxysporum R1 in T6 (corm injury + Fox R1) treatment, Figure S3: Genomic PCR of Fox R1 transformants, Figure S4: Uninfected corm cells under confocal microscope, cells are clearly visible with start granules inside the cells.
