# Supplementary material for: Invasion and Colonization of Pathogenic Fusarium oxysporum R1 in Crocus sativus L. during Corm Rot Disease Progression
Source: J Fungi (Basel). 2022 Nov 25;8(12):1246. doi: 10.3390/jof8121246 (PMC9784501; doi:10.3390/jof8121246)
Supplement: Supplementary file 1 [file jof-08-01246-s001.zip › jof-1830924-supplementary.pdf]

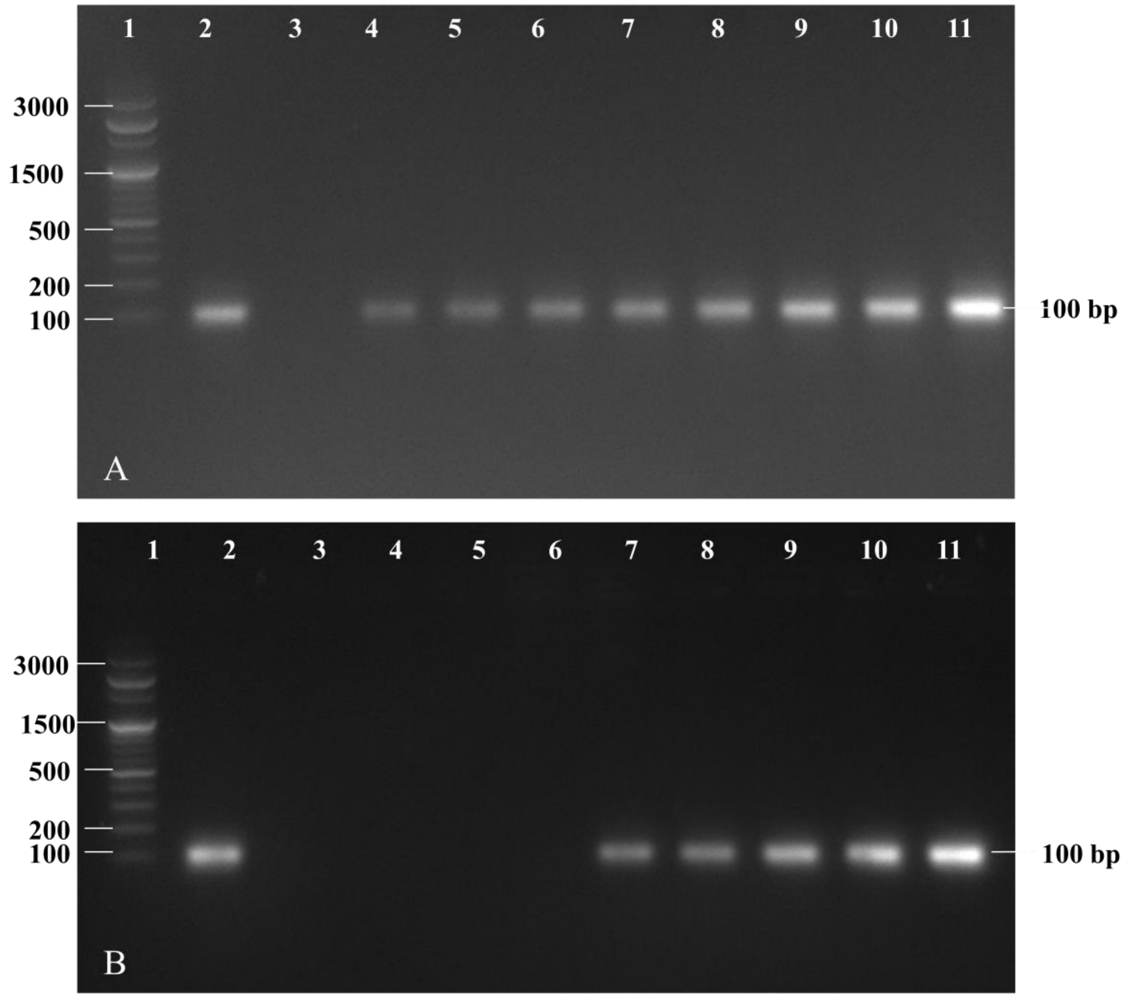

Figure S1 A-B: Gel image showing the amplification of ITS gene of *Fusarium oxysporum* R1 in T4 (corm injury + Fox R1).

A) W4-W11 showing the amplification at 1,3,5,10,15,20,25,30 dpi in corm basal plate and W2 is the positive control (*Fusarium oxysporum* R1 DNA amplified), W3 is the negative control (Corm DNA amplified), W1- 100bp molecular marker

B) W7-W11 showing the amplification in the roots of T4 treatment. At 1,3,5 dpi (W3-W5) no amplification was observed. W2 is the positive control; W3 is the negative control, W1- 100bp molecular marker

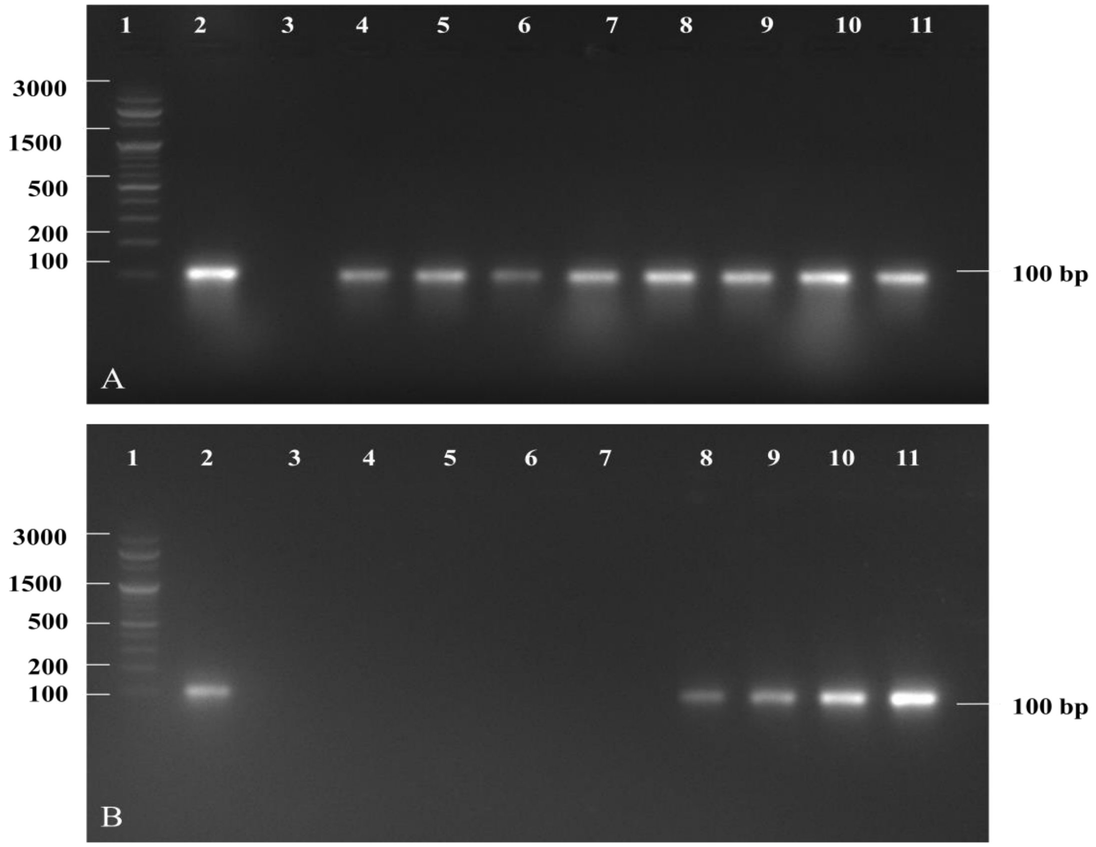

Figure S2 A-B: Gel image showing the amplification of ITS gene of *Fusarium oxysporum* R1 in T6 (corm injury + Fox R1) treatment.

A) W4-W11 showing the amplification at 1,3,5,10,15,20,25,30 dpi in the infected roots and W2 is the positive control; W3 is the negative control, W1- 100bp molecular marker

B) W8-W11 showing the amplification in the corm basal plate of T4 treatment. At 1,3,5, 10 dpi (W4-W7) no amplification was observed in the corm basal plate. W2 is the positive control; W3 is the negative control, W1- 100bp molecular marker

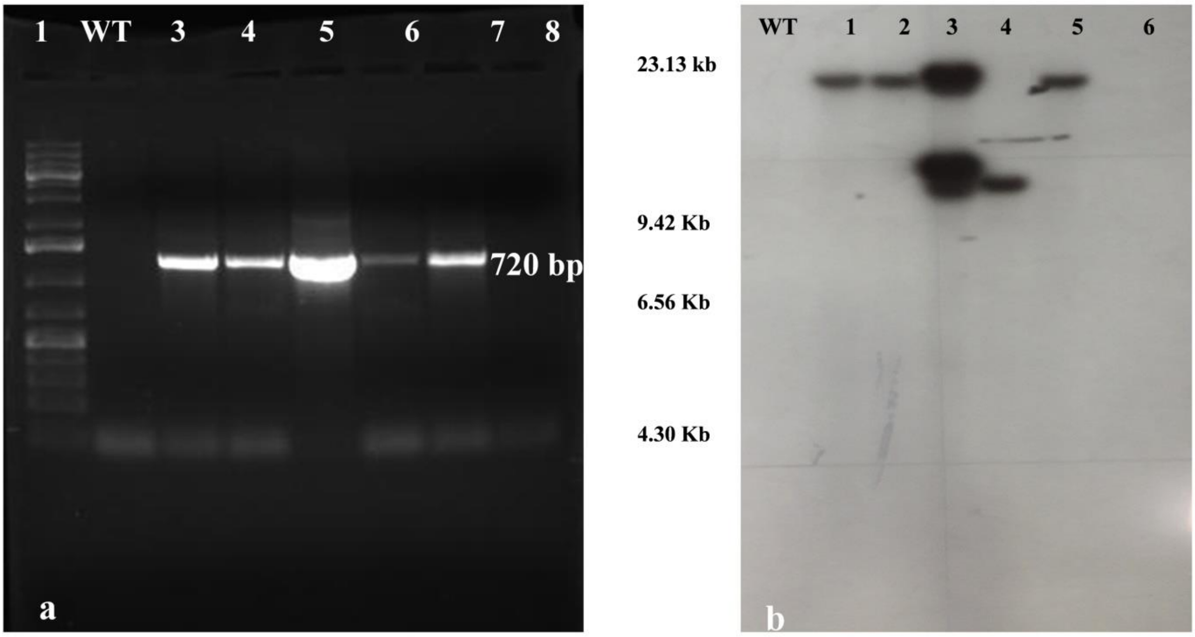

Figure S3: a) Genomic PCR of Fox R1 transformants. W1- 1kb ladder, W2- Wild type, W3 to W8 - transformants

Figure S3b) Southern blot analysis of wild type Fox R1 and randomly selected EGFP tagged Fox R1 transformants.

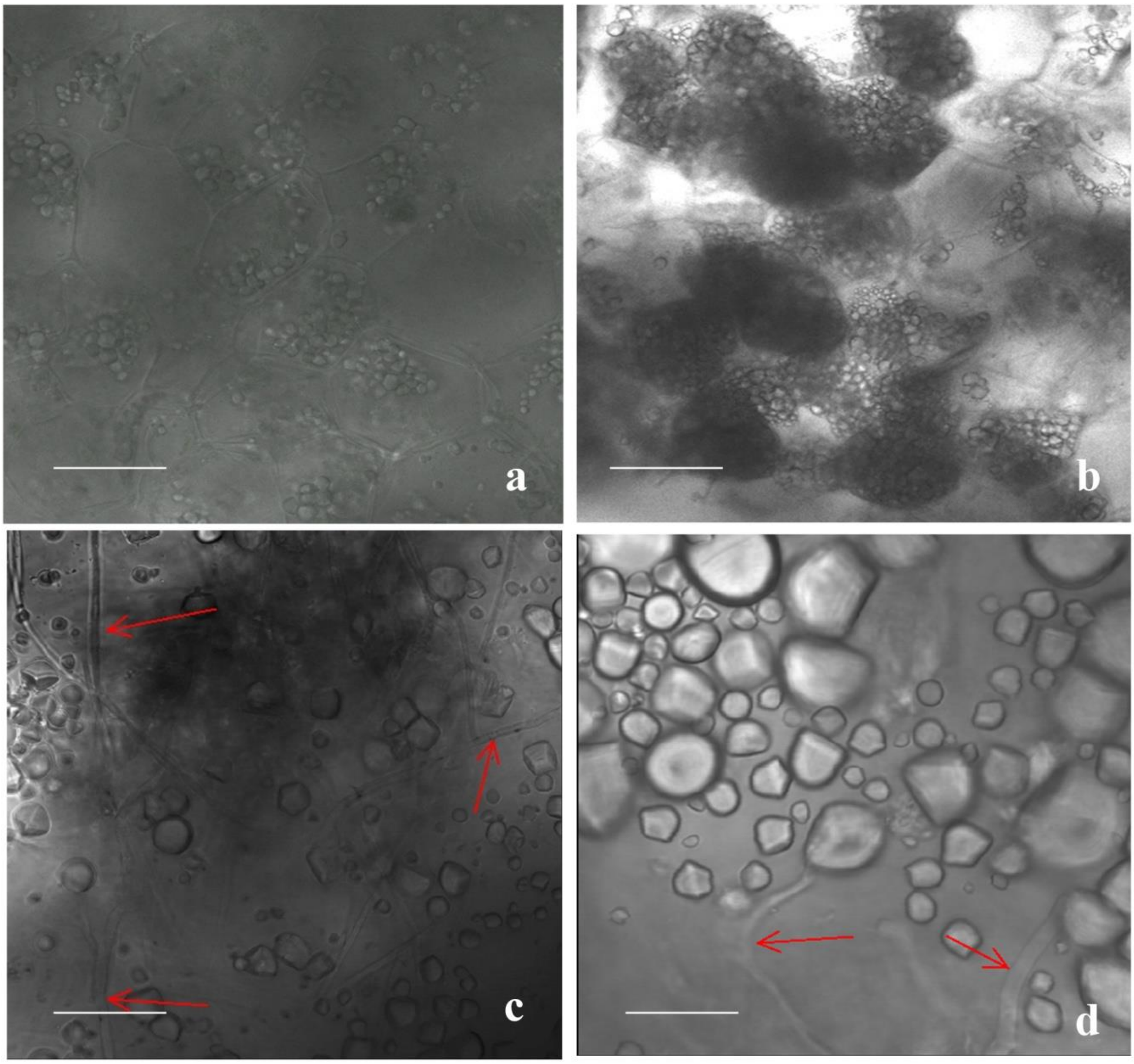

Figure S4: a, b) Uninfected corm cells under confocal microscope, cells are clearly visible with start granules inside the cells. **c, d)** infected corm cells with wild type Fox R1. Each bar represents 10 µm
